# Phenoage and longitudinal changes on transthoracic echocardiography in Alström syndrome: a disease of accelerated ageing?

**DOI:** 10.1007/s11357-023-00959-3

**Published:** 2023-10-02

**Authors:** Leena Patel, Ashwin Roy, Amor Mia B Alvior, Mengshi Yuan, Shanat Baig, Karina V. Bunting, James Hodson, Katja Gehmlich, Janet M Lord, Tarekegn Geberhiwot, Richard P. Steeds

**Affiliations:** 1 Institute of Cardiovascular Sciences, University of Birmingham, Birmingham, UK; 2 Department of Cardiology, University Hospital Birmingham NHS Foundation Trust, Birmingham, Birmingham UK; 3 Research Development and Innovation, University Hospitals Birmingham NHS Foundation Trust, Birmingham, Birmingham UK; 4 Division of Cardiovascular Medicine, Radcliffe Department of Medicine and British Heart Foundation Centre of Research Excellence Oxford, University of Oxford, Oxford, UK; 5 MRC-Versus Arthritis Centre for Musculoskeletal Ageing Research, Institute of Inflammation and Ageing, University of Birmingham, Birmingham, UK; 6 Department of Endocrinology, University Hospital Birmingham NHS Foundation Trust, Birmingham, Birmingham UK; 7 Institute of Metabolism and System Research, University of Birmingham, Birmingham, UK

**Keywords:** Ageing, Echocardiography, Phenoage, Cardiovascular, Rare diseases

## Abstract

Alström syndrome (AS) is an ultra-rare disorder characterised by early-onset multi-organ dysfunction, such as insulin resistance, obesity, dyslipidaemia, and renal and cardiovascular disease. The objective is to explore whether AS is a disease of accelerated ageing and whether changes over time on echocardiography could reflect accelerated cardiac ageing. Cross-sectional measurement of Phenoage and retrospective analysis of serial echocardiography were performed between March 2012 and November 2022. The setting is a single national tertiary service jointly run by health service and patient charity. Forty-five adult patients aged over 16 years were included, 64% were male and 67% of White ethnicity. The median Phenoage was 48 years (interquartile range [IQR]: 35–72) in the 34 patients for whom this was calculable, which was significantly higher than the median chronological age of 29 years (IQR: 22–39, *p*<0.001). Phenoage was higher than chronological age in 85% (*N*=29) of patients, with a median difference of +18 years (IQR: +4, +34). On echocardiography, significant decreases were observed over time in left ventricular (LV) size at end-diastole (average of 0.046 cm per year, *p*<0.001) and end-systole (1.1% per year, *p*=0.025), with significant increase in posterior wall thickness at end-diastole (0.009 cm per year, *p*=0.008). LV systolic function measured by global longitudinal strain reduced (0.34 percentage points per year, *p*=0.020) and E/e’lat increased (2.5% per year, *p*=0.019). Most AS patients display a higher Phenoage compared to chronological age. Cardiac changes in AS patients were also reflective of accelerated ageing, with a reduction in LV size and increased wall thickening. AS may be a paradigm disease for premature ageing.

**Supplementary Information:**

The online version contains supplementary material available at 10.1007/s11357-023-00959-3.

## Introduction

Alström syndrome (AS) is a rare autosomal recessive ciliopathy characterised by childhood retinal dystrophy, neuronal hearing loss and obesity [1]. The phenotype has since been extended to incorporate extreme insulin resistance, type 2 diabetes, dyslipidaemia, accelerated non-alcoholic fatty liver disease and premature renal and cardiovascular disease [2]. The syndrome is caused by loss of function genetic variants in *ALMS1*, a 23-exon gene located on chromosome 2p13 [3]. Infantile cardiomyopathy is the earliest and one of the most frequent manifestations of the syndrome, although the majority survive with apparent complete cardiovascular recovery [4]. However, in a significant proportion, cardiovascular disease either recurs or manifests for the first time in adulthood, with high rates of morbidity and mortality. Both the quality and length of life are reduced in adults with AS, and few survive beyond 50 years [5].

In adult AS subjects, autopsy data demonstrate replacement myocardial fibrosis in non-coronary artery patterns, and diffuse interstitial fibrosis has been detected on cardiovascular magnetic resonance (CMR) by elevation in T1 relaxation and increased extracellular volume [6]. Cardiac fibrosis provokes pathological changes culminating in chamber dilatation, cardiomyocyte hypertrophy and cellular hypertrophy, leading to reduced compliance and accelerated progression to heart failure. Previous studies of AS using echocardiography were limited to a case series that included patients with advanced disease with impaired ejection fraction (EF) [7] and a cross-sectional study of a younger cohort with impaired global longitudinal strain (GLS) [8]. Whilst AS is rare, it offers a model of accelerated cardiomyopathy that reflects disease processes that are common in the ageing population. Given the lack of genotype-phenotype correlation in AS, it is feasible that these fibrotic changes may reflect the long-term impact of obesity, insulin resistance and metabolic syndrome.

Phenoage is a reliable measure of biological ageing calculated using chronological age and nine blood markers representing the functional state of organs [9]. Albumin and alkaline phosphatase (ALP) represent the liver, creatinine the kidney, glucose the pancreas, c-reactive protein (CRP), lymphocyte immune cell percentage and white blood cell count (WCC) represent immune ageing, mean cell volume (MCV) and red cell distribution width (RDW) represent bone marrow age. Phenoage is a reliable predictor for a variety of ageing outcomes, including all-cause mortality, cancer, health span, and physical functioning, but does not incorporate cardiac ageing.

Therefore, the aims of this study were [1] to explore the possibility that AS is a paradigm for accelerated ageing and [2] to explore cardiovascular changes over time in AS.

## Methods

### Ethics

This study was limited to secondary use of information previously collected during normal care (without an intention to use it for research at the time of collection) and is therefore excluded from ethical review according to the UK Health Research Authority decision tool and was registered at our centre as an audit (CARMS-18179).

### Study design

This was an observational, retrospective review of adults (aged over 16 years) with genetically proven AS attending the National Centre for Alström Syndrome at the Queen Elizabeth Hospital Birmingham, UK. Patients attend the centre approximately annually for follow-up assessments, which include blood sampling and transthoracic echocardiography (TTE). All such assessments occurring between March 2012 and November 2022 (the date of data extraction) were identified, and demographic, clinical, biochemical, and cardiovascular data collected were extracted from patient records.

Data were also collected on comorbidities. History of cardiomyopathy was defined as a history of infantile cardiomyopathy [10], presence of myocardial fibrosis identified by late gadolinium enhancement in a non-ischaemic pattern on CMR, and restrictive cardiomyopathy. Ischaemic heart disease was defined as previous myocardial infarction or history of coronary revascularization. Diabetes included a history of both type 1 and 2, regardless of treatment. Renal impairment was defined an estimated glomerular filtration rate (eGFR) <90 mL/min/1.73 m^2^.

### Phenoage

Phenoage was calculated using chronological age and the above nine clinical blood test parameters. Eight of the nine blood markers were included in the routine panel of blood tests performed at every follow-up assessment. However, CRP only became part of this panel in the final year of the study period. As such, Phenoage was only calculated for the most recent assessment for each patient, and those without a CRP were excluded from analyses of Phenoage.

The cohort was divided into groups based on the difference between their chronological and Phenoage at the final scan. Patients where this difference was within ±10 years were classified as having “concordant” Phenoage, with those with a difference of >10 years classified as “discrepant” Phenoage [9].

### Transthoracic echocardiography

Resting transthoracic echocardiography (TTE; ie33 and EPIC, Phillips) was performed by an accredited sonographer (AMA) according to the British Society of Echocardiography minimum dataset [11]. Diastolic function was graded by an experienced cardiologist specialising in echocardiography (RPS) according to current guidelines. Linear internal measurements were obtained from 2D images in the parasternal long axis measured immediately below mitral valve leaflet tips. 2D volumetric measurements were also recorded. There was a focus on parameters considered to be age dependent; a full list of these parameters, along with definitions of the abbreviations used, is reported in Supplementary Table 1.

### Statistical methods

Initially, chronological age and Phenoage at the final scan were compared using Wilcoxon’s Signed Ranks test. This relationship was further assessed using a linear regression model, with the resulting gradient compared to a value of 1, to assess whether Phenoage was increasing at a different rate to chronological age. The Phenoage discrepancy at the final scan was calculated for each patient as Phenoage *minus* chronological age. Patient characteristics, blood markers and TTE parameters were compared between patients with concordant and discrepant Phenoage, using Mann-Whitney *U* tests for continuous variables and Fisher’s exact tests for nominal variables.

Trends over time in blood markers and TTE parameters were then assessed for the whole cohort, as well as by Phenoage discrepancy. Analyses of these factors needed to account for the non-independence of repeated measures on the same patient, which was achieved using two different approaches. The first used generalised estimating equations (GEEs) to adjust for the correlations between repeated measures on the same patient. The second used an “individual regressions” approach, which produced a separate regression model for each patient to estimate the rate of change over time; the gradients of which were extracted and used for analysis. These two approaches applied different weightings to repeated measures on the same patient, with the GEE approach giving greater influence to patients with greater numbers of scans, whilst the individual regressions approach weighted all patients equally. Further details about the two approaches are detailed in the Supplementary Material. Gradients from the models are reported as units per year, % per year, or percentage points (pps) per year, as applicable, along with 95% confidence intervals (95% CIs). Continuous variables are summarised as mean ± standard deviation where approximately normally distributed or as median (interquartile range (IQR)) otherwise; correlation coefficients are reported as Spearman’s rank correlation coefficients (rho). All analyses were performed using IBM SPSS 24 (IBM Corp. Armonk, NY), with *p*<0.05 deemed to be indicative of statistical significance throughout.

## Results

### Cohort characteristics

Of the *N*=49 AS patients treated at the centre, *N*=4 opted for continued follow-up at their local centre after their initial assessment and so were excluded from analysis. The remaining *N*=45 patients were followed up for a median of 6.0 years (IQR: 3.2–9.5), during which they attended a total of 257 follow-up assessments (median: 6 per patient, IQR: 2–8, maximum: 12). The final scan was within one year of data collection in *N*=30 patients; of the remainder, *N*=6 died and *N*=9 were due their next assessment. Patients had median ages of 21 years (IQR: 19–33) and 29 years (IQR: 23–39) at the first and final scan, respectively. Further details of the cohort are reported in Table 1.

**Table 1:** Cohort characteristics

	Whole cohort (*N*=45)	By Phenoage at final scan (*N*=34)		
		*Concordant (N*=11*)*	*Discrepant (N*=23*)*	*p* value
Demographics				
Chronological age (years)				
*First scan*	21 (19–33)	19 (18–24)	21 (18–39)	0.329
*Final scan*	29 (23–39)	27 (21–54)	30 (22–41)	0.348
Gender (% male)	29 (64%)	6 (55%)	15 (65%)	0.709
Ethnicity (% White)	30 (67%)	7 (64%)	17 (74%)	0.692
SBP (at first scan)	130 ± 17	130 ± 21	130 ± 16	0.713
DBP (at first scan)	82 ± 10	83 ± 11	82 ± 10	0.754
Comorbidities*				
Cardiomyopathy	23 (51%)	5 (45%)	11 (48%)	1.000
Ischaemic heart disease	7 (16%)	1 (9%)	2 (9%)	1.000
Hypertension	32 (71%)	6 (55%)	17 (74%)	0.434
Hyperlipidaemia	33 (73%)	6 (55%)	17 (74%)	0.434
Diabetes	37 (82%)	8 (73%)	21 (91%)	0.300
Renal impairment	17 (38%)	0 (0%)	13 (57%)	**0.002**
Physiological markers at final scan**				
Heart rate (bpm)	85 ± 13	80 ± 14	89 ± 9	0.071
Albumin (g/L)	42 ± 4	43 ± 3	40 ± 3	**0.023**
ALP (U/L)	86 (72–108)	71 (56–75)	94 (82–136)	**<0.001**
Creatinine (μmol/L)	97 (78–144)	78 (69–85)	116 (87–148)	**0.002**
Glucose (mmol/L)	6.9 (5.1–12.4)	4.8 (4.0–6.7)	9.9 (5.9–15.9)	**0.002**
HbA1c	53 (39–71)	38 (36–42)	60 (49–77)	**0.002**
CRP (mg/L)	4 (2–16)	2 (1–15)	6 (3–17)	0.133
Lymphocyte (%)	26.9 ± 8.5	28.9 ± 6.3	23.7 ± 8.6	**0.038**
WCC (10^9^/L)	8.7 ± 2.2	9.0 ± 2.7	9.0 ± 2.2	0.854
MCV (Fl)	86.8 ± 6.1	87.5 ± 4.3	87.2 ± 6.4	0.568
RDW (%)	15 (13–16)	14 (13–14)	15 (13–18)	**0.043**

Continuous variables are reported as mean ± standard deviation, or as median (interquartile range), with *p* values from Mann-Whitney *U* tests. Nominal variables are reported as *N* (%), with *p* values from Fisher’s exact tests. Bold *p* values are significant at *p*<0.05

*As diagnosed either at baseline or at any point during follow-up

**Data for the whole cohort were only available for *N*=44 for the blood markers, with the exception of CRP, which was only available in *N*=34

### Phenoage

**Fig. 1:**
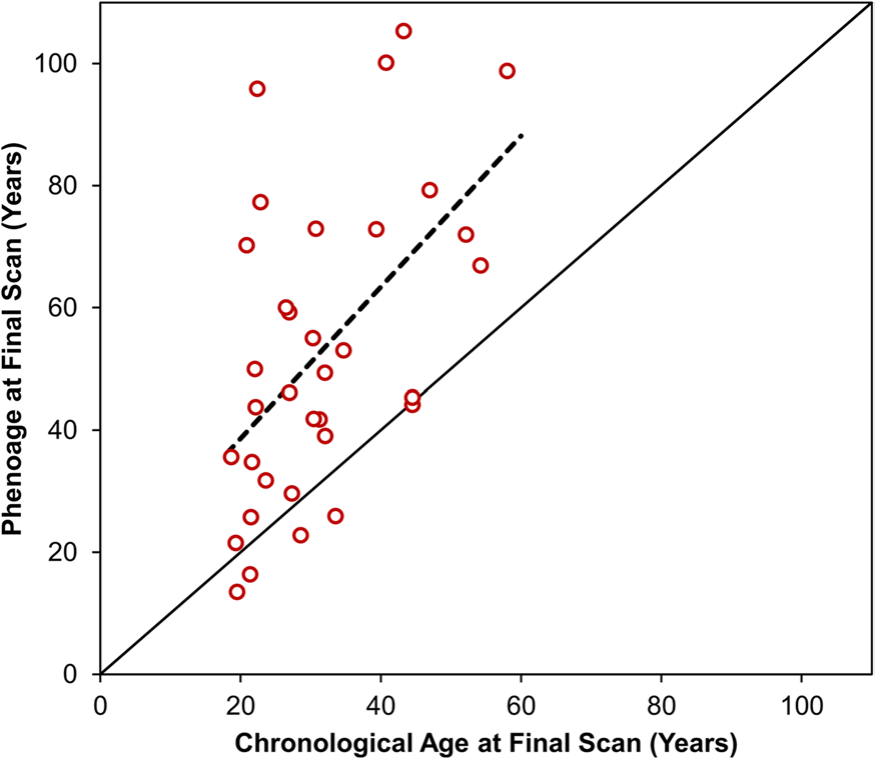
Association between chronological and Phenoage at the final scan. The plot only includes those patients where the Phenoage was calculable at the final scan (*N*=34). The solid line is plotted at *y*=*x*, and the broken line is from a linear regression model

Analyses of Phenoage only included data from the blood tests taken at each patient’s final scan. This resulted in *N*=11 patients being excluded due to lack of CRP values at the time of final scan. Of these, *N*=10 had their final scan prior to CRP being routinely recorded, and *N*=1 did not undergo at blood test at their final scan. The remaining *N*=34 patients had a median Phenoage of 48 years (IQR: 35–72) at their final scan, which was significantly higher than the median chronological age of 29 years (IQR: 22–39, *p*<0.001). Phenoage was higher than chronological age in 85% (*N*=29) of patients, with a median difference of +18 years (IQR: +4, +34), and the largest difference being in a patient with a Phenoage vs. chronological age of 96 vs. 22 years. The association between chronological and Phenoage was assessed using a linear regression approach, visualised in Fig. 1. The gradient of the resulting model, representing the estimated increase in Phenoage per one year of chronological age, was 1.24 (95% CI: 0.54–1.94), which did not differ significantly from a value of 1 (*p*=0.448).

The cohort was divided into subgroups with concordant Phenoage (*N*=11; differences ranging from −8 to +8 years) and discrepant Phenoage (*N*=23; differences ranging from +11 to +74 years, all of whom had a Phenoage greater than chronological age). Comparisons between these two groups found no significant differences in baseline demographics (Table 1). Analysis of comorbidities identified a significant difference in the rates of renal impairment, with a rate of 0% in those with concordant Phenoage, compared to 57% in the discrepant group, with analysis of blood markers at the final scan similarly finding significantly higher creatinine levels in those with discrepant Phenoage (median: 116 vs. 78 μmol/L). Those with discrepant Phenoages also had significantly higher ALP, glucose, HbA1c and RDW levels, as well as significantly lower albumin and lymphocyte counts. Analysis of TTE parameters at the final scan found only LVEDvol 2D to differ significantly between subgroups (*p*=0.035, Supplementary Table 2).

### Trends in blood markers and TTE parameters for the whole cohort

For the cohort of *N*=45 patients, GEE analysis identified significant progression in three blood markers (Table 2), with albumin declining by an average of 0.44 g/L per calendar year, lymphocytes declining by 0.52 pp per year, and creatinine increasing by 2.6% per year. Of the TTE parameters considered, significant decreases were observed in LVEDd and LVESd, with significant increases in LVPWd and LVESvol 2D (Table 2). Analysis using the individual regressions approach in the *N*=32 with data for more than two scans returned similar results, additionally identifying significant increases over time in ALP (2.2% per year), glucose (5.9% per year), GLS total (0.34 pp per year,) and E/e’lat (2.5% per year).

**Table 2:** Trends in blood markers and TTE parameters for the whole cohort

Parameter	GEE approach				Individual regressions approach		
	*No. Pts.*	*No. Scn.*	*Gradient per year (95% CI)*	*p value*	*No. Pts.*	*Gradient per year (95% CI)*	*p value*
Albumin (g/L)	45	251	−0.44 (−0.62, −0.26)	**<0.001**	32	−0.78 (−1.03, −0.53)	**<0.001**
ALP*	45	251	0.7% (−0.8%, 2.1%)	0.387	32	2.2% (0.3%, 4.1%)	**0.027**
Creatinine*	45	251	2.6% (0.4%, 4.7%)	**0.019**	32	3.2% (0.5%, 6.0%)	**0.020**
Glucose*	45	251	0.8% (−1.7%, 3.3%)	0.545	32	5.9% (0.5%, 11.5%)	**0.034**
WCC (10^9^/L)	45	251	−0.03 (−0.11, 0.06)	0.518	32	−0.16 (−0.34, 0.02)	0.075
Lymphocyte (pp**)	45	251	−0.52 (−0.82, −0.22)	**<0.001**	32	−0.60 (−1.08, −0.12)	**0.017**
MCV (Fl)	45	251	0.07 (−0.21, 0.35)	0.642	32	0.03 (−0.29, 0.35)	0.863
RDW (pp**)	45	251	0.04 (−0.05, 0.13)	0.412	32	0.13 (−0.04, 0.30)	0.130
Heart rate (bpm)	45	256	0.35 (−0.25, 0.95)	0.251	32	0.78 (−0.39, 1.96)	0.184
LVIVSd (cm)	45	245	−0.006 (−0.012, 0.001)	0.090	32	−0.004 (−0.012, 0.003)	0.272
LVEDd (cm)	45	248	−0.046 (−0.071, −0.021)	**<0.001**	32	−0.042 (−0.064, −0.020)	**0.001**
LVPWd (cm)	45	245	0.009 (0.002, 0.015)	**0.008**	32	0.016 (0.007, 0.025)	**0.002**
LVESd*	45	231	−1.1% (−2.0%, −0.1%)	**0.025**	32	−1.0% (−1.8%, −0.1%)	**0.031**
EF (pp**)	45	222	−0.03 (−0.56, 0.50)	0.921	32	−0.37 (−0.97, 0.23)	0.215
LVEDvol 2D (ml)	41	133	0.51 (−1.09, 2.10)	0.534	28	0.14 (−1.81, 2.10)	0.880
LVESvol 2D*	41	133	2.8% (0.1%, 5.7%)	**0.042**	28	3.8% (0.3%, 7.4%)	**0.032**
LVEF 2D (pp**)	41	133	−0.44 (−1.13, 0.24)	0.207	28	−1.23 (−2.52, 0.06)	0.061
GLS 4C (pp**)	42	157	0.01 (−0.17, 0.19)	0.913	30	0.09 (−0.20, 0.37)	0.541
GLS total (pp**)	42	156	0.17 (−0.05, 0.39)	0.136	30	0.34 (0.06, 0.63)	**0.020**
LAV*	44	245	0.5% (−1.3%, 2.3%)	0.585	32	1.9% (−0.2%, 4.1%)	0.080
MV E max (cm/s)	45	250	−0.14 (−0.73, 0.44)	0.635	32	0.49 (−0.48, 1.45)	0.310
MV A max (cm/s)	44	242	0.01 (−0.60, 0.62)	0.971	31	−0.03 (−0.74, 0.69)	0.941
MV DT (ms)	43	222	−0.81 (−2.34, 0.72)	0.298	30	0.10 (−5.67, 5.87)	0.973
E/A*	44	241	−0.9% (−2.1%, 0.4%)	0.175	31	0.2% (−1.7%, 2.1%)	0.810
E/E’lat*	45	245	1.4% (−0.1%, 2.8%)	0.064	32	2.5% (0.4%, 4.7%)	**0.019**
E/E’sep	45	235	0.09 (−0.09, 0.26)	0.321	32	0.21 (−0.05, 0.48)	0.113
TDI RV s (cm/s)	43	155	0.07 (−0.10, 0.25)	0.416	31	−0.04 (−0.27, 0.19)	0.712
AV Vmax (cm/s)	45	219	−0.26 (−1.28, 0.76)	0.616	32	0.55 (−0.66, 1.76)	0.362
LVOT Vmax (cm/s)	45	219	−0.08 (−0.83, 0.66)	0.827	32	−0.64 (−1.29, 0.01)	0.054

Analyses were performed using two different approaches. The generalised estimating equation (GEE) approach used GEE models with the timing of the scan test, relative to the first scan, as a covariate, and the stated parameter as the dependent variable. The individual regressions approach first produced separate regression models for each patient with >2 scans (*N*=32) and then took the mean of the resulting gradients, using a one-sample *t*-test, to compare this to a value of zero. Further details of the methodologies used are reported in the Supplementary Material. For both approaches, gradients represent the rate of change per year in each parameter. Bold *p* values are significant at *p*<0.05

*Parameter was log-transformed prior to analysis to improve model fit; the resulting coefficient was then anti-logged and converted into a percentage change per year

**Gradients are reported in percentage points (pps) per year (e.g. a value of 1 would represent an increase from 55 to 56% in one year)

*No. Pts.* number of pts, *No. Scn.* number of scans

### Trends in blood markers and TTE parameters by Phenoage discrepancy

**Fig. 2:**
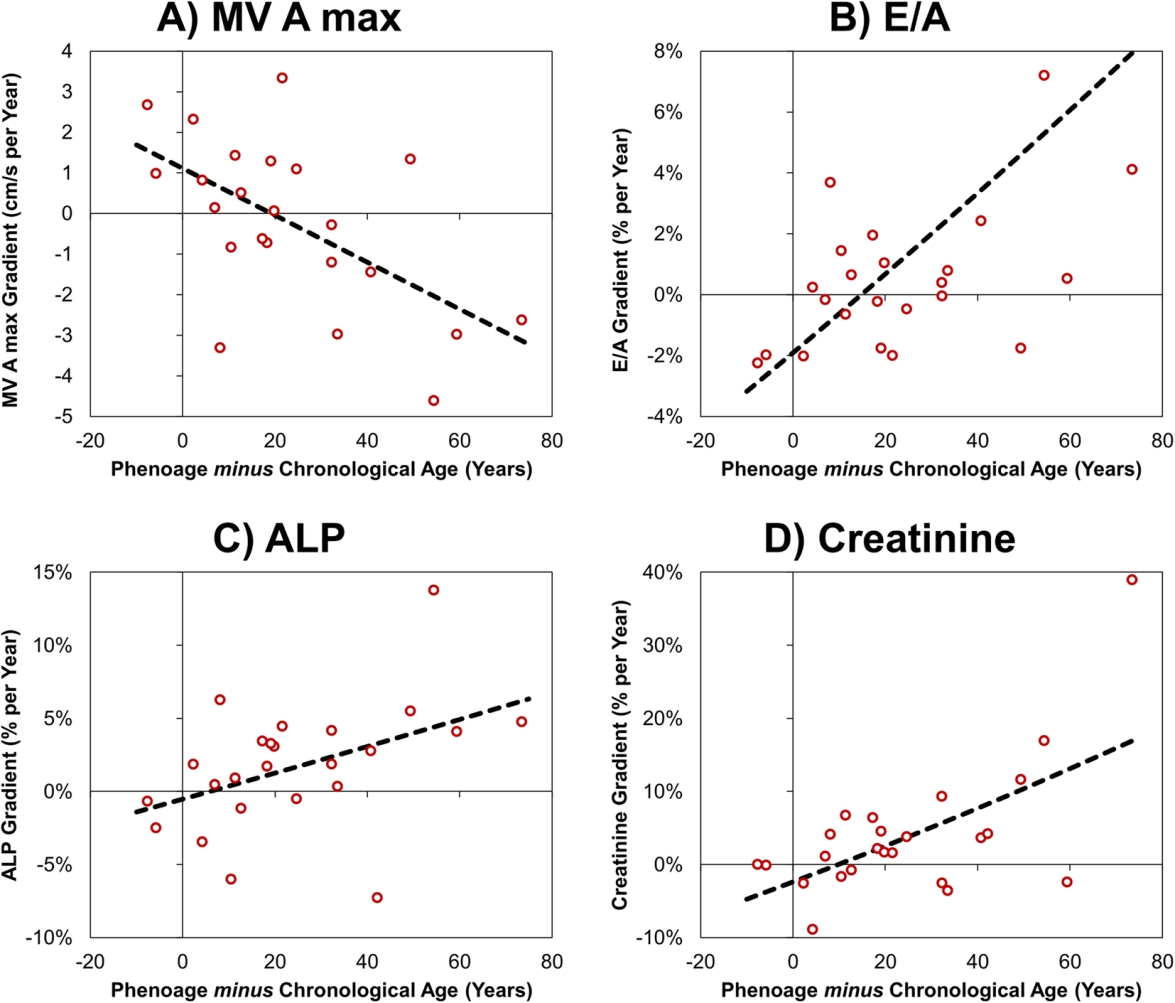
Associations between the magnitude of Phenoage discrepancy and trends in blood markers/TTE parameters. Points represent individual patients, with gradients calculated as per the individual regressions approach (see Table 3) and the magnitude of the Phenoage discrepancy calculated at the final scan. Broken lines are from linear regression models

Both statistical approaches found significant differences in two TTE parameters by Phenoage discrepancy (Table 3). The first was MV A max, which the GEE approach found to be increasing by 1.07 cm/s per year in those with concordant Phenoage, compared to a reduction of 0.28 cm/s per year in those with discrepant Phenoage (*p*=0.023, Supplementary Fig. 1a/b). The individual regressions approach returned similar results, with a negative correlation between the degree of Phenoage discrepancy and MV A max gradient (rho: −0.495, *p*=0.016, Fig. 2a). The second parameter was E/A, for which the GEE analysis identified trends of −2.5% vs. +0.4% per year for concordant vs. discrepant Phenoage (*p*=0.020, Supplementary Fig. 1c/d), with the individual regressions approach finding a significant positive correlation between the degree of Phenoage discrepancy and E/A gradient (rho: 0.451, p=0.031, Fig. 2b).

**Table 3:** Trends in blood markers and TTE parameters by Phenoage discrepancy at the final scan

Parameter	Generalised estimating equation approach					Individual regressions approach		
	*No. Pts.*	*No. Scn.*	*Gradient: concordant subgroup*	*Gradient: discrepant subgroup*	*Interaction p value*	*No. Pts.*	*Spearman’s rho*	*p value*
Albumin (g/L)	34	194	−0.46 (−0.84, −0.07)	−0.40 (−0.61, −0.18)	0.792	24	−0.330	0.115
ALP*	34	194	−1.7% (−4.3%, 1.0%)	1.5% (−0.2%, 3.3%)	0.051	24	0.492	**0.015**
Creatinine*	34	194	0.7% (−1.6%, 3.1%)	2.9% (−0.3%, 6.3%)	0.283	24	0.424	**0.039**
Glucose*	34	194	−0.1% (−3.7%, 3.6%)	1.1% (−2.3%, 4.5%)	0.638	24	0.392	0.058
WCC (10^9^/L)	34	194	0.00 (−0.10, 0.10)	0.02 (−0.11, 0.14)	0.843	24	−0.345	0.098
Lymphocyte (pp**)	34	194	−0.57 (−1.09, −0.05)	−0.54 (−0.91, −0.17)	0.925	24	−0.109	0.613
MCV (Fl)	34	194	−0.23 (−0.56, 0.10)	0.15 (−0.26, 0.56)	0.163	24	0.102	0.636
RDW (pp**)	34	194	−0.02 (−0.09, 0.05)	0.03 (−0.09, 0.16)	0.506	24	0.101	0.639
Heart rate (bpm)	34	194	1.05 (−0.09, 2.19)	0.37 (−0.32, 1.05)	0.314	24	−0.128	0.552
LVIVSd (cm)	34	186	−0.004 (−0.017, 0.009)	−0.007 (−0.016, 0.001)	0.670	24	−0.289	0.171
LVEDd (cm)	34	188	−0.053 (−0.103, −0.002)	−0.025 (−0.054, 0.003)	0.357	24	−0.034	0.875
LVPWd (cm)	34	186	0.008 (−0.006, 0.022)	0.008 (−0.001, 0.017)	0.998	24	−0.350	0.094
LVESd*	34	179	−1.7% (−3.3%, −0.2%)	−0.5% (−1.7%, 0.7%)	0.224	24	0.075	0.728
EF (pp**)	34	172	0.97 (0.12, 1.83)	−0.29 (−0.99, 0.41)	**0.025**	24	0.183	0.391
LVEDvol 2D (mL)	32	102	1.40 (−1.99, 4.78)	0.99 (−1.10, 3.08)	0.842	23	0.123	0.578
LVESvol 2D*	32	102	7.6% (0.1%, 15.7%)	3.0% (−0.7%, 6.9%)	0.294	23	0.021	0.925
LVEF 2D (pp**)	32	102	0.00 (−1.10, 1.10)	−0.57 (−1.43, 0.29)	0.423	23	0.118	0.593
GLS 4C (pp**)	32	124	−0.20 (−0.48, 0.07)	0.06 (−0.14, 0.27)	0.548	24	−0.227	0.286
GLS total (pp**)	32	123	0.01 (−0.26, 0.28)	0.20 (−0.11, 0.51)	0.357	24	−0.284	0.178
LAV*	33	187	−2.5% (−6.1%, 1.2%)	1.1% (−1.1%, 3.4%)	0.099	24	−0.346	0.098
MV E max (cm/s)	34	190	−0.52 (−1.66, 0.63)	0.07 (−0.61, 0.76)	0.387	24	0.070	0.744
MV A max (cm/s)	33	184	1.07 (0.23, 1.91)	−0.28 (−1.09, 0.53)	**0.023**	23	−0.495	**0.016**
MV DT (ms)	32	172	−0.99 (−3.24, 1.26)	−0.74 (−3.02, 1.54)	0.879	23	0.301	0.162
E/A*	33	184	−2.5% (−4.3%, −0.6%)	0.4% (−1.1%, 1.9%)	**0.020**	23	0.451	**0.031**
E/E’lat*	34	186	1.4% (−1.5%, 4.4%)	1.1% (−0.4%, 2.6%)	0.857	24	−0.127	0.554
E/E’sep	34	177	0.02 (−0.27, 0.31)	0.12 (−0.13, 0.37)	0.617	24	−0.047	0.828
TDI RV s (cm/s)	32	117	0.04 (−0.12, 0.19)	0.12 (−0.17, 0.42)	0.623	23	−0.350	0.101
AV Vmax (cm/s)	34	166	−0.63 (−1.83, 0.57)	−0.22 (−1.77, 1.32)	0.684	24	0.076	0.725
LVOT Vmax (cm/s)	34	166	1.02 (0.29, 1.74)	−0.27 (−1.41, 0.87)	0.063	24	−0.193	0.366

For the generalised estimating equation (GEE) approach, only *N*=34 patients for whom Phenoage was calculable at the final scan were included. GEE models were produced with the stated parameter as the dependent variable and the timing of the scan/blood test, subgroup (discrepant vs. concordant Phenoage) and an interaction term as covariates. The “gradients” represent the rate of change per year in each parameter and are reported with the 95% confidence interval; the “interaction” is the *p* value for the interaction term in the model, representing a comparison between the gradients in the two subgroups. The individual regressions approach first produced separate regression models for each patient with >2 scans for whom Phenoage was calculable at the final scan (*N*=24). The correlations between the resulting gradients and the Phenoage discrepancy (Phenoage minus chronological age) were then assessed using Spearman’s rho. Further details of the methodologies used are reported in the Supplementary Material. Bold *p* values are significant at *p*<0.05

*Parameter was log-transformed prior to analysis to improve model fit; the resulting gradient was then anti-logged and converted into a percentage change per year

**Gradients are reported in percentage points (pps) per year (e.g. a value of 1 would represent an increase from 55 to 56% in one year)

*No. Pts.* number of pts, *No. Scn.* number of scans

The GEE approach additionally identified a significant difference in the gradients for EF, which was increasing by 0.97 pp per year in those with concordant Phenoage, but decreasing by 0.29 pp per year in those where this was discrepant (*p*=0.025, Supplementary Fig. 1e/f). However, the individual regressions analysis found no significant association between the degree of Phenoage discrepancy and the EF gradient, with the effect being in the opposite direction (rho: 0.183, *p*=0.391).

The individual regressions approach also identified significant positive correlations between the degree of Phenoage discrepancy and gradients in both ALP (*p*=0.015, Fig. 2c) and creatinine (*p*=0.039, Fig. 2d), implying that these markers saw more pronounced increases over time in those with more discrepant Phenoage; similar trends were observed in the GEE analyses.

## Discussion

In this longitudinal study of the largest cohort of AS patients published, we identified accelerated ageing as a defining feature, with most adults displaying an advanced Phenoage compared with chronological age. In the cohort, 85% of patients with Phenoage data exhibited a higher Phenoage than chronological age, indicating impaired structural and functional status of organs at an earlier age than expected. This raises the possibility that AS is a paradigm disease for accelerated ageing, with potential to act as a model for multi-organ dysfunction that occurs with obesity, insulin resistance, type 2 diabetes, and dyslipidaemia. The dysregulation and impairment of underlying biological processes may therefore act as a mechanism of premature ageing, consistent with the reduced life expectancy observed in AS.

There was no evidence to suggest that Phenoage increased at a faster rate than chronological age in this cohort; instead, the discrepancy appeared to be present at referral to the service and to persist thereafter.

Of patients displaying accelerated ageing, significant changes were detected in blood markers relating to renal and liver dysregulation. Serum creatinine was significantly increased and positively correlated with Phenoage discrepancy, indicating reduced creatinine clearance and glomerular filtration rates (GFR) representing renal injury [12, 13]. Reduced GFR and elevated serum creatinine represent a decline in renal function, which decreases with age [14]. Trends of elevated ALP and Phenoage discrepancy indicate hepatic involvement contributing an accelerated ageing. AS patients display chronic kidney disease, hepatic fibrosis and renal impairment from early ages, which progress linearly with age [15, 16]. Renal impairment may be linked to *ALMS1* playing a role in sodium reabsorption, affecting renal sodium transporters, further impacting endocytosis and impairing renal function [17, 18].

In addition, elevated Hba1c levels were identified in the discrepant Phenoage group, consistent with the phenomenon of impaired glucose tolerance seen in AS [19]. *ALMS1* mutations can alter glucose homeostasis, by reducing glucose transporter expression (GLUT4) and relocalisation in cells, leading to aberrant glucose transport, altering insulin trafficking [20, 21]. Reduced insulin sensitivity is seen in the elderly population, supporting the concept of accelerated metabolic ageing in AS [22].

Low serum albumin may also reflect ageing and associations have been demonstrated between reduced serum albumin and elevated mortality in elderly cohorts [23]. Low albumin further served as a prognostic marker for other impairments such as inflammation and malabsorption. Albumin misfolding and structural modifications with age may impair its homeostatic functions, contributing to an inflammatory state [24]. This is supported by a reduced lymphocyte count, portraying a compromised immune system in those with discrepant Phenoages [25]. The collective changes in serum parameters are congruous with the multi-organ involvement in AS.

LV dimensions including LVEDd and LVESd decreased significantly over time, denoting reduced LV size. This is consistent with published longitudinal studies demonstrating marked reduction in LVEDd with physiological ageing [26]. Increased LVPWd is consistent with a thicker LV wall, seen in physiological ageing [27]. LV wall thickening may be due to cardiomyocyte hypertrophy, rather than increased cardiomyocyte number, as reduced cardiomyocyte number is associated with ageing [28]. Hypertension which elevates LV end-diastolic pressure can lead to cardiomyocyte thickening as a compensatory mechanism to preserve cardiac output [29].

Dividing the cohort into concordant and discrepant Phenoage, a significant positive correlation in E/A ratio with the degree of Phenoage discrepancy suggests increased LV pressure, impacting diastolic function, often impaired with age [30]. Diastolic dysfunction may occur in AS due to increased LV stiffening from fibrosis [31] related to excess collagen deposition, impairing relaxation [32]. Changes in contractility could be linked to the primary role of *ALMS1* in cell cycle regulation and extracellular matrix production [33]. AS patients with mutant *ALMS1* may consequently display altered cellular proliferation, leading to cardiomyocyte senescence and thus altering diastolic function [34]. This is further reflected by a reduction in MV A max in the discrepant Phenoage cohort, supporting LV stiffening and diastolic dysfunction. Variations in these parameters are expected to alter with age, supporting the concept of premature cardiovascular ageing in AS. Whilst ageing can be reflected with cardiac parameters, they do not impact Phenoage.

### Strengths and limitations

This study has several strengths, chief amongst which are the large sample size for a rare disease and long follow-up time with a median of six years. Data were also available from regular assessments for blood markers and TTE parameters, allowing for a range of factors to be analysed. These two statistical approaches used generally gave consistent results, providing additional validation to these findings.

However, there were also some limitations that need to be considered when interpreting the findings. Primarily, CRP was not routinely tested during the study period; therefore, it was only possible to calculate Phenoage at patients’ final follow-up assessments. This resulted in a quarter of patients being excluded from Phenoage analysis and also precluded analyses of longitudinal trends in Phenoage. Additionally, Phenoage was assessed at the end of the study period and compared to previous follow-up. Consequently, the findings of the analysis cannot be interpreted as indicating the ability for Phenoage discrepancy to predict future trends in blood markers or TTE parameters.

In addition, there was a large variation in the numbers of scans per patient due to different dates patients were initially referred to the service, with most patients having a follow-up assessment within one year of the end of the study. During the COVID-19 pandemic, numbers were reduced as patients had either died or been lost to follow-up before the end of the study period. As such, the truncation of follow-up in these patients may have introduced some degree of selection bias. Whilst the two statistical approaches generally returned consistent results, there were some inconsistencies, specifically when assessing trends in EF by Phenoage. Consequently, it is not possible to reliably draw conclusions where the two approaches had inconsistent findings.

## Conclusion

AS is a disease model of accelerated ageing, with most patients displaying an increased biological Phenoage compared to chronological age. A wide discrepancy in Phenoage was present from entry into the clinical service, suggesting that multi-organ dysfunction and an altered ageing trajectory occur at an early age, with the difference then stabilising with age. Accelerated cardiac ageing in AS was also found on longitudinal assessment using TTE, with progressive LV reduction in size, increase in LV wall thickness and reduction in mitral early filling with increase in atrial filling. Despite recapitulating cardiac re-modelling in AS, cardiac parameters have less impact on Phenoage discrepancy. The findings open up new possibilities for treatment via the use of repurposed drugs able to slow or reverse the rate of biological ageing [35].

### Supplementary information

ESM 1 Supplementary Figure 1 – Patient trajectories in TTE parameters by Phenoage discrepancy. Points and solid lines represent trajectories for individual patients. Separate plots are produced for those with concordant and discrepant Phenoages when calculated at the final scan. Broken lines are trend lines from generalised estimating equation models, as described in Table 3 (PNG 1991 kb)

High resolution image (TIFF 1054 kb)

ESM 2 (DOCX 43 kb)
