# Supplementary figures and images for: Phenoage and longitudinal changes on transthoracic echocardiography in Alström syndrome: a disease of accelerated ageing?
Source: GeroScience. 2023 Oct 2;46(2):1989–99. doi: 10.1007/s11357-023-00959-3 (PMC10828353; doi:10.1007/s11357-023-00959-3)

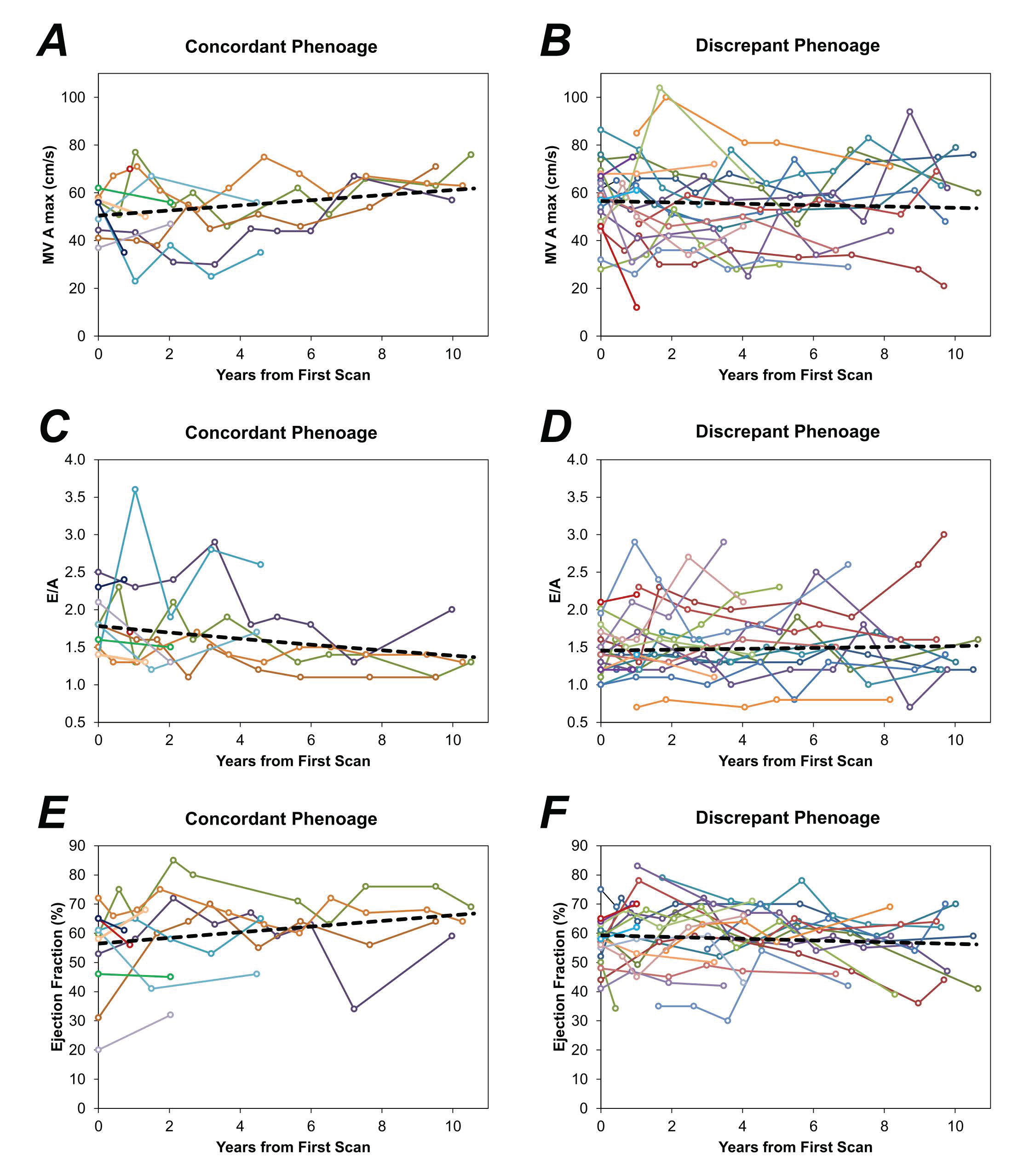

Supplement: Supplementary file 1 — Supplementary Figure 1 – Patient trajectories in TTE parameters by Phenoage discrepancy. Points and solid lines represent trajectories for individual patients. Separate plots are produced for those with concordant and discrepant Phenoages when calculated at the final scan. Broken lines are trend lines from generalised estimating equation models, as described in Table 3 (PNG 1991 kb) [file 11357_2023_959_Fig3_ESM.png]

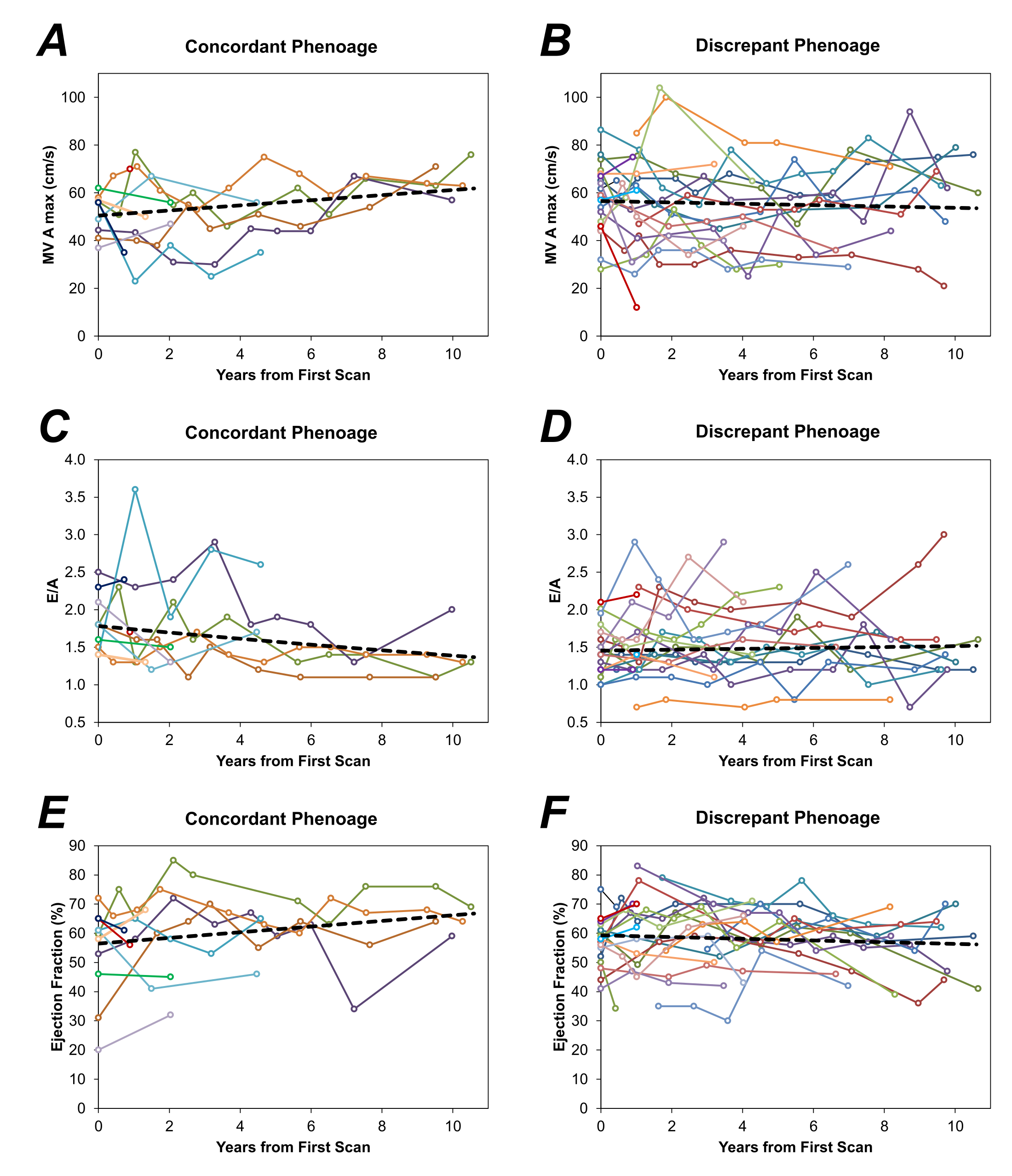

Supplement: Supplementary file 2 — High resolution image (TIFF 1054 kb) [file 11357_2023_959_MOESM1_ESM.tiff]
