# Supplementary material for: Phenoage and longitudinal changes on transthoracic echocardiography in Alström syndrome: a disease of accelerated ageing?
Source: GeroScience. 2023 Oct 2;46(2):1989–99. doi: 10.1007/s11357-023-00959-3 (PMC10828353; doi:10.1007/s11357-023-00959-3)
Supplement: Supplementary file 3 — (DOCX 43 kb) [file 11357_2023_959_MOESM2_ESM.docx]

**SUPPLEMENTARY MATERIAL**

**Statistical methods – Additional details of approaches to modelling longitudinal trends**

Analyses of trends over time in blood markers and TTE parameters needed to account for the non-independence of repeated measures on the same patient. This was performed using two different approaches, namely the generalized estimating equations (GEE) approach, and the individual regressions approach. Further details of these two approaches are described below.

The GEE approach used GEE models, with the scan number as the within-subject variable, and assumed an AR (1) correlation structure to account for the within-patient correlation. These models were first used to estimate the trends in blood markers and TTE parameters for the cohort as a whole. As such, the parameter of interest was set as the dependent variable, with the time from the first scan to the current scan entered as a covariate. The models were then extended to additionally include the Phenoage subgroup and an interaction term. The resulting models estimated the gradients over time for the concordant and discrepant Phenoage subgroups separately, with the interaction term representing a comparison between these two gradients.

The analysis was also performed using an individual regressions approach. This first produced a separate linear regression model for each patient, with the parameter of interest as the dependent variable, and the time from the first scan to the current scan as a covariate. Only those patients with more than two scans were included in this analysis, as analysis of two data points was deemed insufficient to reliably estimate long-term trends. In order to estimate the trends in blood markers and TTE parameters for the cohort as a whole, the mean of the resulting gradients was calculated across all included patients and compared to a value of 0 using a one-sample t-test. Associations between the gradients over time and Phenoage discrepancy were then assessed. Due to the smaller sample size included in the individual regressions analysis, this analysis treated the degree of Phenoage discrepancy as a continuous variable (i.e. Phenoage *minus* chronological age), rather than dichotomising into subgroups. Spearman's rank correlation coefficients (rho) were then calculated to quantify the association between the degree of Phenoage discrepancy at the final scan and the gradients in blood markers and TTE parameters from the individual regression models.

Prior to analyses using both approaches, the goodness-of-fit of the GEE models for the cohort as a whole were assessed by examination of the residuals. Where these were skewed, indicating a non-linear association, log_10_-transformations were applied to the affected variables to improve model fit. These log_10_-transformed variables were then used in all subsequent analysis. For all analyses, gradients are reported as the rate of increase per year in the stated parameter. For parameters that were measured as a percentage (e.g. ejection fraction), the gradients represented percentage point (pp) per year increases. For parameters that were log_10_-transformed for analysis, the resulting coefficients were anti-logged, such that gradients were reported as percentage increases per year.

***Supplementary Table 1 – List of TTE parameters***

| **Parameter** | **Name** |
| --- | --- |
| LVIVSd (cm) | Left ventricular intraventricular septum end diastole |
| LVEDd (cm) | Left ventricular end-diastolic dimension |
| LVPWd (cm) | Left ventricular posterior wall thickness end diastole |
| LVESd (cm) | Left ventricular end-systolic dimension |
| EF (%) | Ejection fraction |
| LVEDvol 2D (ml) | Left ventricular end-diastolic volume |
| LVESvol 2D (ml) | Left ventricular end-systolic volume |
| LVEF 2D (%) | Left ventricular ejection fraction modified by Simpson’s rule |
| GLS 4C (%) | Global longitudinal strain 4 chamber |
| GLS Total (%) | Global longitudinal strain total calculated on 2D speckle training |
| LAV (ml) | Left atrial volume by biplane area-length |
| MV E max (cm/s) | Mitral valve early filling |
| MV A max (cm/s) | Mitral valve atrial filling |
| MV DT (ms) | Mitral valve deceleration time |
| E/A | Ratio between E-wave and A-wave |
| E/E'lat | Lateral ratio |
| E/E'sep | Septal ratio |
| TDI RV s (cm/s) | Tissue doppler Imaging right ventricle |
| AV Vmax (cm/s) | Aortic valve gradient |
| LVOT Vmax (cm/s) | Left ventricular outflow tract |

***Supplementary Table 2 – TTE parameters at the final scan***

|  | **Whole Cohort** | | **By Phenoage at Final Scan** | | | |
| --- | --- | --- | --- | --- | --- | --- |
| **Parameter** | **N** | **Statistic** | ***N*** | ***Concordant*** | ***Discrepant*** | ***p-Value*** |
| LVIVSd (cm) | 45 | 1.0 (0.9-1.1) | 34 | 1.0 (0.9-1.1) | 1.0 (0.9-1.1) | 0.834 |
| LVEDd (cm) | 45 | 4.65 ± 0.76 | 34 | 5.05 ± 0.79 | 4.58 ± 0.67 | 0.074 |
| LVPWd (cm) | 45 | 1.01 ± 0.13 | 34 | 1.04 ± 0.11 | 1.03 ± 0.11 | 0.562 |
| LVESd (cm) | 45 | 2.9 (2.5-3.9) | 34 | 3.5 (2.9-4.2) | 3.1 (2.5-3.9) | 0.276 |
| EF (%) | 44 | 61 (46-69) | 34 | 61 (46-65) | 59 (43-69) | 0.941 |
| LVEDvol 2D (ml) | 27 | 100 ± 34 | 19 | 126 ± 29 | 91 ± 34 | **0.035** |
| LVESvol 2D (ml) | 27 | 38 (29-63) | 19 | 56 (36-78) | 34 (29-51) | 0.151 |
| LVEF 2D (%) | 27 | 56 ± 10 | 19 | 53 ± 14 | 57 ± 8 | 0.641 |
| GLS 4C (%) | 37 | -18 ± 4 | 28 | -19 ± 4 | -18 ± 4 | 0.674 |
| GLS Total (%) | 36 | -17 ± 3 | 27 | -17 ± 2 | -16 ± 4 | 0.699 |
| LAV (ml) | 42 | 41 (31-59) | 31 | 40 (34-60) | 42 (25-63) | 0.916 |
| MV E max (cm/s) | 44 | 85 ± 18 | 33 | 88 ± 19 | 85 ± 16 | 0.620 |
| MV A max (cm/s) | 42 | 57 (46-69) | 32 | 56 (47-70) | 61 (44-69) | 0.843 |
| MV DT (ms) | 38 | 166 ± 32 | 29 | 164 ± 48 | 166 ± 27 | 0.506 |
| E/A | 42 | 1.5 (1.2-1.7) | 32 | 1.5 (1.3-2.0) | 1.4 (1.2-1.6) | 0.632 |
| E/E'lat | 43 | 7.0 (6.0-9.0) | 32 | 6.5 (5.5-8.0) | 7.3 (6.0-8.5) | 0.376 |
| E/E'sep | 39 | 10.7 ± 3.9 | 29 | 11.2 ± 5.4 | 10.7 ± 3.5 | 0.687 |
| TDI RV s (cm/s) | 40 | 12.8 ± 2.7 | 30 | 13.0 ± 2.2 | 12.7 ± 3.2 | 0.371 |
| AV Vmax (cm/s) | 43 | 117 ± 19 | 32 | 116 ± 14 | 117 ± 23 | 0.889 |
| LVOT Vmax (cm/s) | 42 | 87 ± 17 | 31 | 85 ± 16 | 86 ± 19 | 0.901 |

*Data are reported as mean ± standard deviation, or as median (interquartile range), with p-values from Mann-Whitney U tests. Bold p-values are significant at p<0.05.*
